# Effect of Interpersonal Interaction on Festinating Gait Rehabilitation in Patients with Parkinson’s Disease

**DOI:** 10.1371/journal.pone.0155540

**Published:** 2016-06-02

**Authors:** Hirotaka Uchitomi, Ken-ichiro Ogawa, Satoshi Orimo, Yoshiaki Wada, Yoshihiro Miyake

**Affiliations:** 1 Department of Computational Intelligence and Systems Science, Tokyo Institute of Technology, Yokohama, Kanagawa, Japan; 2 Department of Neurology, Kanto Central Hospital, Setagaya, Tokyo, Japan; 3 Department of Rehabilitation, Nissan Tamagawa Hospital, Setagaya, Tokyo, Japan; University of Toronto, CANADA

## Abstract

Although human walking gait rhythms are generated by native individual gait dynamics, these gait dynamics change during interactions between humans. A typical phenomenon is synchronization of gait rhythms during cooperative walking. Our previous research revealed that fluctuation characteristics in stride interval of subjects with Parkinson’s disease changed from random to 1/*f* fluctuation as fractal characteristics during cooperative walking with the gait assist system Walk-Mate, which emulates a human interaction using interactive rhythmic cues. Moreover, gait dynamics were relearned through Walk-Mate gait training. However, the system’s clinical efficacy was unclear because the previous studies did not focus on specific gait rhythm disorder symptoms. Therefore, this study aimed to evaluate the effect of Walk-Mate on festinating gait among subjects with Parkinson’s disease. Three within-subject experimental conditions were used: (1) preinteraction condition, (2) interaction condition, and (3) postinteraction condition. The only difference between conditions was the interactive rhythmic cues generated by Walk-Mate. Because subjects with festinating gait gradually and involuntarily decreased their stride interval, the regression slope of stride interval as an index of severity of preinteraction festinating gait was elevated. The regression slope in the interaction condition was more gradual than during the preinteraction condition, indicating that the interactive rhythmic cues contributed to relieving festinating gait and stabilizing gait dynamics. Moreover, the gradual regression slope was carried over to the postinteraction condition, indicating that subjects with festinating gait have the potential to relearn stable gait dynamics. These results suggest that disordered gait dynamics are clinically restored through interactive rhythmic cues and that Walk-Mate may have the potential to assist therapists in more effective rehabilitation.

***Trial Registration*:** UMIN Clinical Trials Registry UMIN000012591

## Introduction

**Fig 1:**
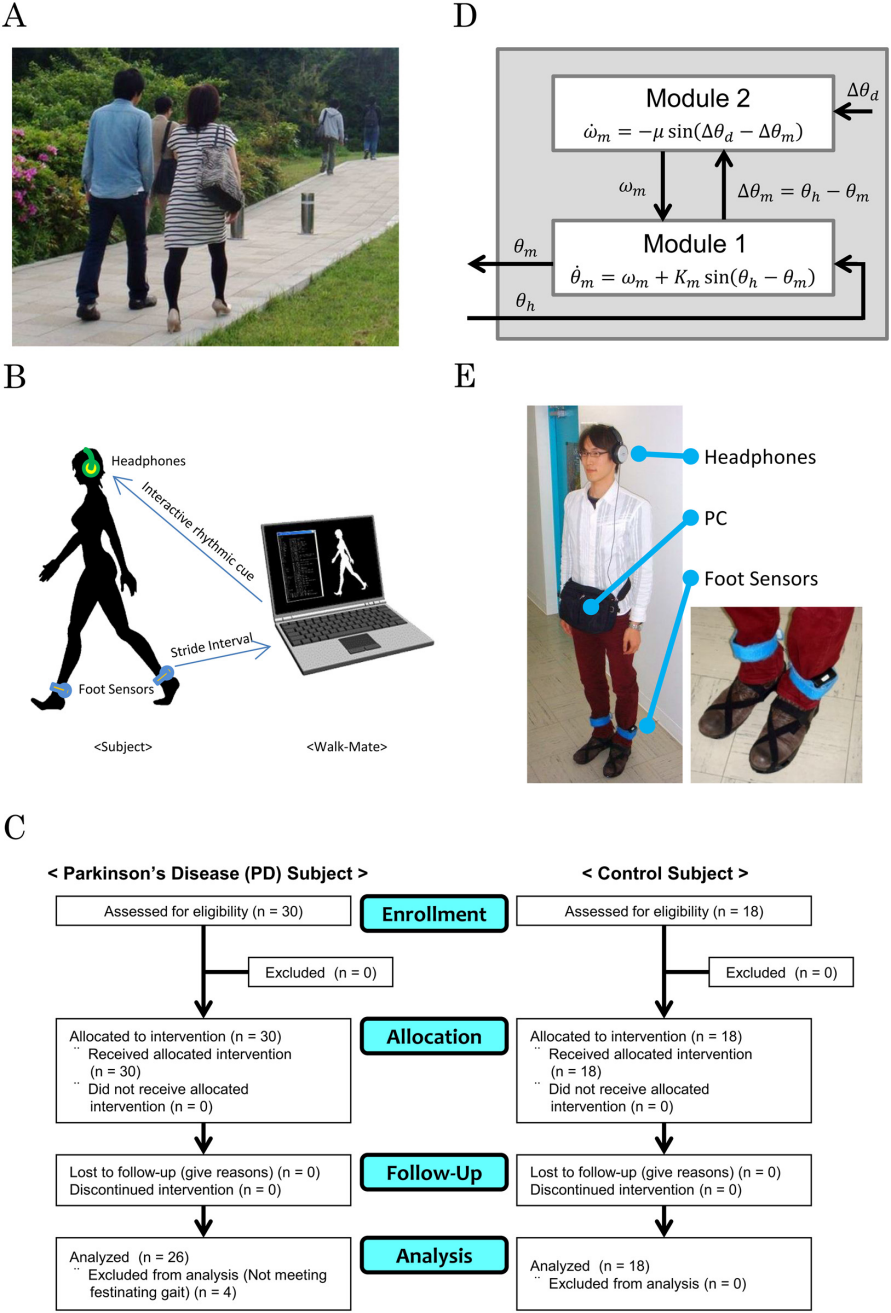
Interpersonal interaction between the gait of two humans. (A) Cooperative walking. Two humans walk together with interpersonally synchronized gait rhythms. (B) Emulation of interpersonal interaction using an interactive rhythmic cue. The Walk-Mate system emulates the gait rhythm of one of the two humans. The system’s gait rhythm is generated through interpersonal interaction with the human gait rhythm. (C) CONSORT flowchart of the current study, based on the website http://www.consort-statement.org/. (D) Model of rhythm generation. The model has a hierarchical structure, in which two dynamics were mutually constrained. The model details are described in [8]. (E) Experimental setup. Subjects wear foot sensors and headphones. The foot sensors are pressure sensors for gathering data on the time stamp of each step in the subject’s gait, which are sent to a personal computer (PC). Headphones then provide the interactive rhythmic cue generated in the PC based on the rhythm generation model.

Synchronization of human gait rhythms often occurs when humans walk together during “cooperative walking” (Fig 1A). Considerable research has been devoted to the relationship between interpersonal synchronization and gait dynamics [1–3]. The gait rhythms during interpersonal synchronization differ from when the subject walks alone. Rhythm generation in human gait has a native intrapersonal dynamic [4,5] and this changes through an interpersonal interaction with the intrapersonal dynamics of another human [6,7]. Interpersonal interaction between a therapist and a patient is widely used for gait rehabilitation because such an interpersonal interaction contributes to improving gait abnormality and relearning gait dynamics.

Our previous study revealed that intrapersonal gait dynamics changed in interpersonal interaction of gait rhythms between humans [9]. Specifically, 1/*f* fluctuation appeared in the stride interval of both healthy subjects and those with Parkinson’s disease (PD) [10] when they walked cooperatively with the Walk-Mate system [11–15]. Here, 1/*f* fluctuation in the stride interval means the power of the fluctuation (spectrum density) is inversely proportional to frequency in the time series data, and often indicates fractal characteristics and self-similarity. Walk-Mate emulates the interpersonal synchronization of gait rhythms between humans through interpersonal interaction using interactive rhythmic cues [4,5,16]. Therefore, 1/*f* fluctuation in stride interval is one of the properties indicating healthy gait states [16–18]. In contrast, this fluctuation property did not appear in the stride of subjects when they walked to a fixed-tempo rhythmic cue without any interpersonal interaction [4,9,19]. Another of our previous studies clarified that gait rehabilitation with interpersonal interaction contributed to relearning human intrapersonal gait dynamics [20].

Previous work has shown that (1) interpersonal interaction is significant for restoring healthy intrapersonal gait dynamics, and (2) that the intrapersonal gait dynamics are not only restored but are also relearned. Further, the relearned intrapersonal gait dynamics carry over after discontinuation of the interpersonal interaction. Thus, interpersonal interaction is necessary for restoring intrapersonal gait dynamics in gait rehabilitation.

However, in previous work, the potential for clinical application was unclear because the studies did not focus on a specific gait rhythm symptom among subjects with PD [9,20]. When considering clinical application for gait rehabilitation, it is important to focus on a concrete gait symptom that is directly applicable to a specific gait disorder and to clarify whether such a treatment method is effective for that gait symptom.

Festinating gait is a specific clinical gait symptom among patients with PD and is a gait disorder with a malfunction of the gait rhythm generation system. In disabilities such as PD, degeneration of the basal ganglia causes movement, timing, and rhythm malfunction [21–23] because human gait rhythm generation involves a distributed and interactive network that depends on the basal ganglia [24]. Thus, festinating gait is regarded as a disorder of the gait rhythm generation system rather than a musculoskeletal system disorder. A PD patient with festinating gait shows a gait speed that gradually and involuntarily accelerates; the patient must stop walking or they will eventually fall over [25,26]. Thus, the symptom is inhibited sustainable gait because of the monotonically accelerating gait speed. It is considered to be possible to clarify a range of clinical effect of interpersonal interaction, by applying interpersonal interaction to this gait disorder,

The aim of this study was to clarify the clinical application of interpersonal interaction on the intrapersonal gait dynamics of gait rhythm generation. Specifically, this study focused on festinating gait as representing gait disorders with a malfunction of the gait rhythm generation system among patients with PD. The goal was to quantify the degree of festinating gait based on stride interval and then evaluate the effect of interpersonal interaction on festinating gait. The interpersonal interaction in gait rhythm was implemented by the Walk-Mate system, which emulates the interpersonal interaction of gait rhythms between humans using interactive rhythmic cues. We also investigated how the festinating gait of patients with PD changed and whether the effect carried over following interaction with the Walk-Mate system.

## Materials and Methods

### Subjects

Thirty idiopathic PD subjects (14 men and 16 women) with normal hearing and no evidence of dementia participated. The mean ± standard deviation (SD) ages and disease durations of the subjects with PD were 74.9 ± 7.10 and 6.05 ± 4.21 years, respectively. Subjects’ modified Hoehn and Yahr disease stage (HY) [27–29] was 2.77 ± 0.450; parts 2 and 3 of the Unified Parkinson’s Disease Rating Scale (UPDRS-2 and UPDRS-3) [28,30,31] were 10.0 ± 4.22 and 22.7 ± 7.35, respectively. Details are shown in Table 1. Because UPDRS-2 and UPDRS-3 were not performed with one subject for clinical reasons, this subject was excluded from these values. All subjects with PD were taking dopaminergic medication and continued medication during the study. They exhibited festinating gait during a prescreening interview with a physician.

**Table 1:** Characteristics of Subjects with Parkinson’s Disease.

PD Subject	Age	Sex	Disease Duration	HY^†^	UPDRS^††^	
					Part 2	Part 3
1	68	male	13	3	6	29
2	75	male	12	3	15	31
3	75	male	6	3	10	22
4	83	female	12	3	10	26
5	78	female	9	3	12	29
6	76	female	9	3	15	37
7	82	female	7	3	14	14
8	80	female	7.5	3	13	24
9	73	female	6.5	2.5	5	22
10	76	male	0.9	3	12	23
11	72	female	6	3	10	28
12	52	female	4	2	6	13
13*	72	female	2	2.5	11	15
14	79	female	3	2.5	3	21
15	75	male	5	2.5	6	16
16	77	male	1	2.5	5	21
17*	67	female	5	3	5	9
18	72	female	3	3	8	19
19	79	female	3	3	8	22
20	79	male	3	3	9	21
21	92	male	2	3	22	29
22	73	female	5	2	8	18
23	61	female	0.5	3	13	38
24	82	male	3	2.5	12	21
25^‡^	78	male	0.8	3	–	–
26	77	male	2	2	7	14
27	71	male	0.2	3	15	26
28*	71	female	4	2	12	25
29*	77	male	7	4	13	33
30	74	male	3	2	5	11

* Subjects who did not show festinating gait.

^†^ HY is modified Hoehn and Yahr disease stage.

^††^ UPDRS is Unified Parkinson’s Disease Rating Scale.

^‡^ UPDRS was not performed for subject 25 due to clinical reasons.

The control group was 18 (12 men and 6 women) healthy, age-matched, nonhospitalized participants who did not have any gait disorder and who also had normal hearing and no dementia. Control subjects were 70.6 ± 3.07 years.

The study was approved by the Kanto Central Hospital Ethics Committee in Japan (The trial protocol and the TREND checklist for this study are available as supporting information; see S1, S2 and S3 Files). Recruitment of all PD and healthy control subjects took place within a 24-month period. Written informed consent was obtained from all subjects before participation and they were compensated for participating. The individuals appearing in the figures of this manuscript have given written informed consent to publish the details of this study.

### Conditions and Tasks

There were three experimental conditions: preinteraction, interaction, and postinteraction. We investigated whether interactive rhythmic cues influenced the festinating gait of subjects with PD and whether the effect was carried over following the cues. In the interaction condition, each of the subjects with PD walked while listening to the interactive rhythmic cues generated by the Walk-Mate system, details of which are described in the Experimental System subsection below (Fig 1B). During the pre- and postinteraction conditions, subjects walked alone without any audible cues. The interaction cues were the only variable that differed between conditions.

The experimental task was to walk along 80 m of the hospital corridor. Each of the subjects with PD walked this route twice during the experiment. The first time was a preliminary task, and the second was the experimental task for gait measurement. During the preliminary task, the subject was equipped with an experimental system and walked the corridor to become accustomed to the experimental environment. Their total walk time was measured and used to set the conditions for the experimental task. After the preliminary walk, the experiment was carried out and data were collected for analyses.

During the experiment task, the three conditions (preinteraction, interaction, and postinteraction) occurred sequentially. Time to cover the distance during each condition was determined based on the total time measured during the preliminary task. Subjects were instructed to walk along the hospital corridor along a straight 80 m line while wearing the experimental setup; cues were provided during the middle third of the walk and stopped for the last third. Participants were not instructed regarding the type of cues that would be provided or exactly when the cues would start and stop.

In the event that subjects with PD had a significant festinating gait that caused them to fall or suddenly stop during the cue sequence, or based on the clinical judgment of a medical doctor, they carried out only the preinteraction and interaction conditions and were excluded from the postinteraction condition. In the event that the postinteraction condition was stopped early by a subject, the preinteraction and interaction conditions only were analyzed and the postinteraction condition was excluded from analyses. The control subjects only performed the normal gait condition, which was the same as the preinteraction condition; these data were used as the baseline in analyses.

The study CONSORT flowchart is shown in Fig 1C. It was determined that the minimum sample size was 12 in each experimental group, based on the hypothetical significance level, effect size, and statistical power. The number of required groups was set at four based on the experimental conditions and tasks; the required significance level (alpha) was set at 0.05, the effect size was set at 0.5, and statistical power was set at 0.8 based on previous studies [32,33].

No follow-up survey was used after completion of the experimental tasks. The experimental conditions and tasks were registered with the University Hospital Medical Information Network (UMIN) Center (www.umin.ac.jp), UMIN Clinical Trials Registry (UMIN-CTR) Identifier UMIN000012591.

### Experimental System

The Walk-Mate system generated the interactive rhythmic cues and interpersonally interacted with the subject’s individual gait rhythm using rhythmic cues [15]. The interactive rhythmic cues interpersonally synchronized with the gait rhythm of subjects with PD, with presentation of the cues (Fig 1B).

The Walk-Mate rhythm generator system incorporated a rhythm generator with a hierarchical structure consisting of two modules (Fig 1D). Module 1 interpersonally synchronized the gait rhythm and cued a rhythm based on the mutual entrainment mechanism. Module 1 is described as:

$${\dot{\theta }}_{m}=\omega_{m}+K_{m}\text{sin}\left(\theta_{h}-\theta_{m}\right),$$ (1)

where *θ*_*m*_ represents the phase variable of the Walk-Mate system and *ω*_*m*_ denotes the natural frequency of the system. Here *θ*_*m*_ is an integer multiple of 2*π*, *θ*_*h*_ represents the phase variable of the subject’s gait rhythm, which is estimated based on their stride interval, and *K*_*m*_ (>0) designates a given coupling constant. This module was implemented using a nonlinear oscillator.

Module 2 controlled the phase difference between the gait and cue rhythms to mate the phase difference *Δθ*_*m*_ (= *θ*_*h*_ − *θ*_*m*_) with a target phase difference *Δθ*_*d*_. Module 2 is described as:

$${\dot{\omega }}_{m}=-\mu \text{sin}\left(\Delta \theta_{d}-\Delta \theta_{m}\right),$$ (2)

where *μ* denotes a control gain. Here, when the target phase difference *Δθ*_*d*_ was a positive value, module 2 shifted a natural frequency of the nonlinear oscillator to generate a cue rhythm so that the cue rhythm became the phase delay for the gait rhythm, depending on an absolute value of the target phase difference. The parameters of the rhythm generator model in the Walk-Mate system were set based on previous studies [9,20]. These equations can be applied to both the right and left legs using a phase shift of *π*. In this study, the values of *K*_*m*_, *μ*, and *Δθ*_*d*_ were set to 0.5, 0.32, and 0.2, respectively. The model details have been described previously [34].

The hierarchical model for interpersonal rhythm generation has been supported by neuroscience. The human gait is influenced by rhythm adjustment depending on the central pattern generator of the spinal cord and by feedback controls in the cerebellum and brain stem [35,36]. Moreover, behavioral experimental results on cooperative finger tapping [37,38] and human research on the dual process model [39] provide evidence of a hierarchical model.

Regarding the physical system, the Walk-Mate system consists of a personal computer (PC) laptop (CF-W5, Panasonic, Japan), headphones (HP-RX500, Victor, Japan), and pressure sensors (OT-21BP-G, Ojiden, Japan), which are attached to the bottom of the subject’s shoes, a radio transmitter connecting the pressure sensors, and a radio receiver connecting the sensors and the laptop (Fig 1E). The control algorithm of the rhythm generator was coded by using C Programming Language. The pressure sensors measured the time series of time stamps of steps in the subject’s gait. The radio transmitters received the data and transmitted to the laptop through the radio receiver. The algorithm running in real time on the laptop calculated the interval sequence to generate an oscillatory rhythm based on the nonlinear oscillator and received the time series about the subject’s gait. The oscillatory rhythm generated by the algorithm was provided to the subject in the interaction condition by alternately using two cues whose sounds consisted of the square wave of 10 ms and whose tones were F5 and C5, through the headphones. These processes were carried out in parallel in real time. The laptop also saved the time series of the steps with a sampling period of 10 ms, to calculate the subject’s stride interval and analyze it offline following data collection.

### Data Analysis

The stride interval of each subject’s gait was provided based on the calculation of the time series of time stamps of steps in their gait. The subject’s stride interval *T*_*h*_ is described as:

$$T_{h}\left(i\right)=t_{h}\left(i+1\right)-t_{h}\left(i\right),$$ (3)

where *t*_*h*_(*i*) is the *i*-th time stamp of the step in the subject’s gait.

Subsequently, the rate of change of stride interval (*β*) was evaluated to quantify the degree of festinating gait and to determine the influence on the festinating gait of the interactive rhythmic cue generated by the Walk-Mate system. Rate *β* was derived by calculating the coefficient of regression slope of stride interval:

$$\beta =\frac{n\sum_{i=1}^{n}t_{h}\left(i\right)T_{h}\left(i\right)-\sum_{i=1}^{n}t_{h}\left(i\right)\sum_{i=1}^{n}T_{h}\left(i\right)}{n\sum_{i=1}^{n}t_{h}{}^{2}\left(i\right)-{\left\{\sum_{i=1}^{n}t_{h}\left(i\right)\right\}}^{2}},$$ (4)

where *n* is the final time stamp of the step in the subject’s gait. Festinating gait is the gait symptom in which speed involuntarily accelerates and thus the stride interval of a festinating gait gradually decreases. The feature of festinating gait was the focus here and its degree was quantified by evaluating rate *β*. A low value of *β* meant that the subject increased the severity of their festinating gait. A *β* value near zero meant that the subject had a gait with stable speed uniformity.

The targeted subjects for this experiment had been diagnosed with PD and had festinating gait as one symptom. Therefore, even if a physician had given a subject with PD a diagnosis of having festinating gait before the experiment, the patient was excluded from the study if they did not show festinating gait symptom on the day of the study. Specifically, the current study established a threshold value for rate *β*. Only when a subject with PD had *β* of less than the threshold (control) value in the preinteraction condition was the subject included in the evaluation. If it was difficult for a subject with PD to carry out the postinteraction condition because of the judgment of a medical doctor or the judgment of the subject with PD himself/herself, only the preinteraction and interaction conditions were analyzed.

We compared the four experimental conditions: preinteraction, interaction, postinteraction, and control (identical to the preinteraction condition). The analysis parameters were the rate of change of stride interval (*β*), the mean, and the coefficient of variance in stride interval, which were calculated using MATLAB^®^ (MathWorks, Natick, MA). Comparisons were statistically analyzed using one-way factorial analyses of variance and post hoc tests were made using the Tukey–Kramer method. Statistical analyses were performed using SPSS^®^ (SPSS Inc., Chicago, IL).

To clarify the relationship between the index value of disease severity classification from diagnosis and the influence of interactive rhythmic cues on the festinating gait, we also divided subjects with PD into two groups whose index values of disease severity were high and low compared with the median of the index value. Experimental results were then compared between the preinteraction and interaction conditions for each group. The reported *p*-values are for two-sided tests. In addition, we report the Pearson’s product-moment correlation coefficients between the index value (disease severity classification from diagnosis) and interactive rhythmic cues on the festinating gait to consider the scope of relationship between these measures.

## Results

**Fig 2:**
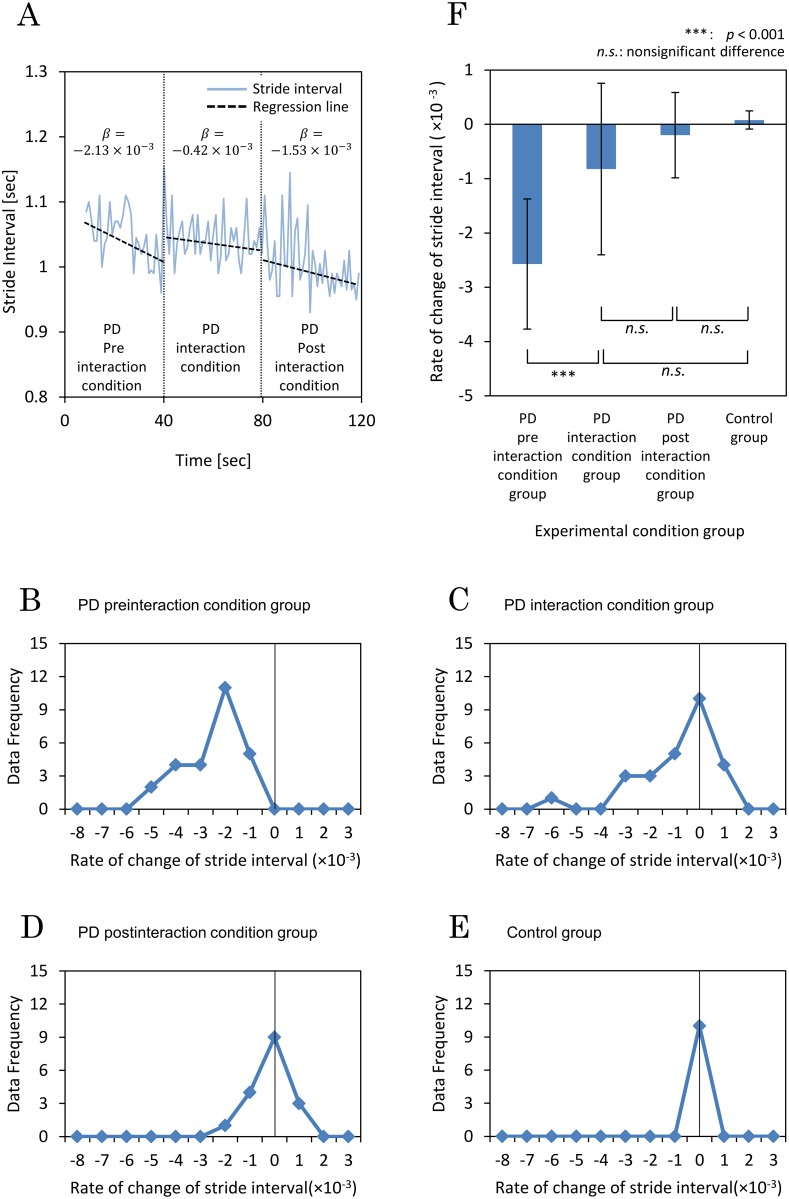
Study results. (A) Example of time series data for stride interval of a subject with Parkinson’s disease (PD) (solid lines). In the preinteraction condition, the subject walked without the interactive rhythmic cues. The subject showed festinating gait in this stage, seen as the slope (*β* = –2.13 × 10^−3^) of the stride interval regression line (dashed line) inclined downward to the right. However, during the interaction condition, when the Walk-Mate system gave the subject interactive rhythmic cues, the regression slope (*β* = –0.42 × 10^−3^) appeared flatter than during the preinteraction condition. This indicates that gait in the interaction condition was more stable, or of a more constant speed. Then, in the postinteraction condition, the flatter regression slope (*β* = –1.53 × 10^−3^) was carried over, demonstrating that the PD subject’s festinating gait was improved by interacting with the cues even after the cues were removed. (B) Data frequency of rate *β* during the preinteraction condition. (C) Data frequency of rate *β* during the interaction condition. (D) Data frequency of rate *β* during the postinteraction condition. (E) Data frequency of rate *β* among the control subjects. (F) Mean and standard deviation of rate *β* during all experimental conditions. Using the Tukey–Kramer method, the two-sided *p*-values were ***p<0.001; **p<0.01; *p<0.05; *n*.*s*. indicates a nonsignificant difference.

Fig 2A shows an example of the experimental results. Here, a subject with PD walked along the corridor in a straight line during the three experimental conditions (preinteraction with silence, interaction with interactive rhythmic cues, and postinteraction again with silence). In each state shown in Fig 2A, the dashed line represents the regression line of the time series data for stride interval and *β* is the slope of the regression line (i.e., the rate of change for stride interval). A smaller *β* means a more decreased stride interval and a specific *β* decrease indicates the degree of festinating gait. The rate *β*s in the subjects with PD during the interaction and postinteraction conditions were larger and nearer to zero than during the preinteraction condition, indicating improvement in the festinating gait.

The threshold value of rate *β* was set to 1.00 × 10^−3^ in reference to the experimental results for the control subjects. Only results from subjects with PD whose rate *β* was less than this threshold value were included for analyses. In the preinteraction condition, 26 of 30 subjects with PD showed festinating gait. Thus, these 26 subjects with PD were included in the preinteraction and interaction analyses. Nine of the subjects with PD stopped walking during the third condition based on the judgment of a medical doctor or the PD subject himself/herself. Thus, only the remaining 17 of 26 subjects with PD were included in the postinteraction condition analyses to evaluate a carry-over effect. Eighteen subjects took part in the control analyses. Therefore, the statistical analyses compared four groups: (a) 26 subjects with PD in the preinteraction condition group; (b) 26 subjects with PD in the interaction condition group; (c) 17 subjects with PD in the postinteraction condition group; and (d) 18 control subjects (The dataset of time series of the stride interval is available in figshare.com (DOI: 10.6084/m9.figshare.3219655).

Fig 2B–2F and Tables 2 and 3 show the distribution and statistical results of the rates of change in stride interval (*β*) for each of the four experimental groups. The experimental conditions affected rate *β* (*F*(3,83) = 24.7, *p*<0.001). Post hoc results show that rate *β* in the preinteraction condition group (*M* ± *SD* = –2.57 × 10^−3^ ± 1.20 × 10^−3^) was significantly smaller compared with the interaction condition group (*M* ± *SD* = –0.823 × 10^−3^ ± 1.58 × 10^−3^) (*p*<0.001), the postinteraction condition group (*M* ± *SD* = –0.200 × 10^−3^ ± 0.786 × 10^−3^) (*p*<0.001), and the control group (*M* ± *SD* = 0.0803 × 10^−3^ ± 0.167 × 10^−3^) (*p*<0.001). However, rate *β* in the interaction condition group was not significantly different from the postinteraction condition (*p* = 0.3) and the control condition (*p* = 0.06). Rate *β* in the control condition was also not significantly different from the PD postinteraction condition (*p* = 0.9).

**Table 2:** Omnibus Analysis and Post Hoc Analyses of Change Ratio of Stride Interval for PD and Healthy Subjects.

Evaluation Index	Experimental Condition				Analysis of Variance	
	PD subjects			Healthy subjects	*F* value, *p* value	
	Preinteraction	Interaction	Postinteraction	Silent		
Change Ratio of Stride Interval [×10^−3^]	–2.57 ± 1.20	–0.823 ± 1.58	–0.200 ± 0.786	0.0803 ± 0.167	*F*(3, 83) = 24.7, *p*<0.001	***

***p<0.001.

**Table 3:** Significance Test for Change Ratio of Stride Interval for PD and Healthy Subjects.

	PD Subjects				Healthy Subjects
	Preinteraction		Interaction	Postinteraction	Silent
PD Subjects					
Preinteraction	–		–	–	–
Interaction	*p*<0.001	***	–	–	–
Postinteraction	*p*<0.001	***	*p* = 0.3	–	–
Healthy Subjects					
Silent	*p*<0.001	***	*p* = 0.06	*p* = 0.9	–

***p<0.001.

**Fig 3:**
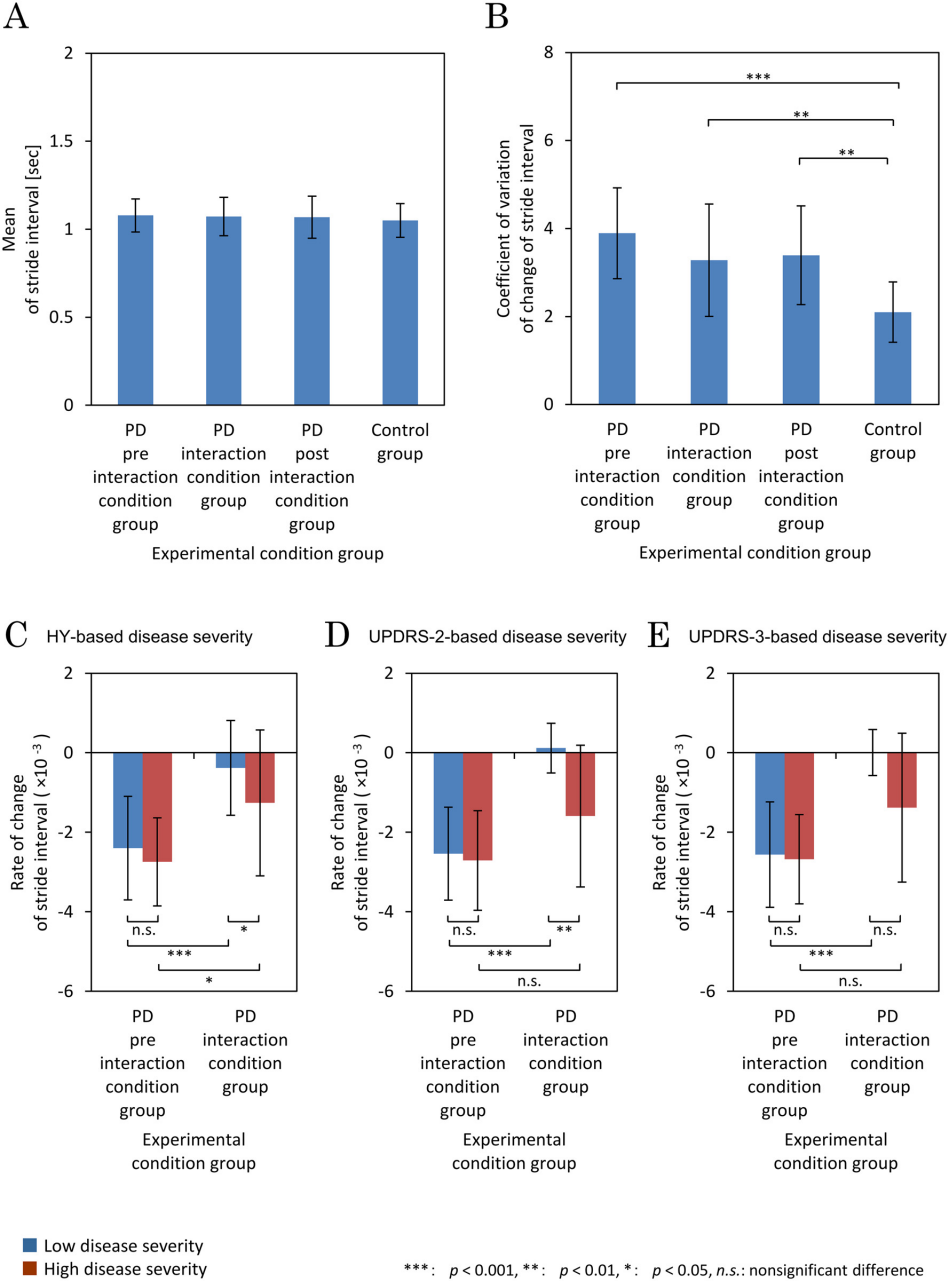
Supplementary analyses. (A) Mean and standard deviation of stride interval in each condition with the reported *p*-values: ***p<0.001; **p<0.01; *p<0.05; *n*.*s*. indicates a nonsignificant difference. (B) Mean and standard deviation of the coefficient of variation for stride interval in all experimental conditions. (C) The rate of change of stride interval (*β*) of the low and high disease severity groups in each of the preinteraction and interaction conditions. Disease severity was based on the modified Hoehn and Yahr stage (HY). (D) Rate *β* of the low and high disease severity groups in the preinteraction and interaction conditions. Disease severity was based on Part 2 of the Unified Parkinson’s Disease Rating Scale (UPDRS-2). (E) Rate *β* of the low and high disease severity groups in the preinteraction and interaction conditions. Disease severity was based on UPDRS-3.

For supplemental analyses, Fig 3A and Table 4 summarize the results of the comparisons of the mean stride interval among the four groups. The experimental conditions did not affect the mean stride interval (*F*(3,83) = 0.271, *p* = 0.8). There was no significant difference between the means in the preinteraction condition group (*M* ± *SD* = 1.08 × 10^−3^ ± 0.0940 × 10^−3^), the interaction condition group (*M* ± *SD* = 1.07 × 10^−3^ ± 0.109 × 10^−3^), the postinteraction condition group (*M* ± *SD* = 1.07 × 10^−3^ ± 0.119 × 10^−3^), and the control group (*M* ± *SD* = –1.05 × 10^−3^ ± 0.0957 × 10^−3^) (*p*>0.05 for all comparisons).

**Table 4:** Omnibus Analysis and Post Hoc Comparisons of Stride Interval for PD and Healthy Subjects.

Evaluation Index	Experimental Condition				Analysis of Variance	
	PD subjects			Healthy subjects	*F* value, *p* value	
	Preinteraction	Interaction	Postinteraction	Silent		
Mean	1.08 ± 0.0940	1.07 ± 0.109	1.07 ± 0.119	1.05 ± 0.0957	*F*(3, 83) = 0.271, *p* = 0.8	
Coefficient of Variation	3.89 ± 1.03	3.28 ± 1.28	3.39 ± 1.12	2.10 ± 0.687	*F*(3, 83) = 10.1, *p*<0.001	***

***p<0.001.

In addition, Fig 3B and Tables 4 and 5 summarize the results of the coefficient of variation of stride interval between the four experimental groups. The experimental conditions affected the coefficient of variation (*F*(3,83) = 10.1, *p*<0.001). Post hoc results show that the coefficient of variation of the control condition group (*M* ± *SD* = 2.10 × 10^−3^ ± 0.687 × 10^−3^) was significantly smaller than the preinteraction condition group (*M* ± *SD* = 3.89 × 10^−3^ ± 1.03 × 10^−3^) (*p*<0.001), the interaction condition group (*M* ± *SD* = 3.28 × 10^−3^ ± 1.28 × 10^−3^) (*p*<0.01), and the postinteraction condition group (*M* ± *SD* = 3.39 × 10^−3^ ± 1.12 × 10^−3^) (*p*<0.01). However, there were nonsignificant differences between the preinteraction, interaction, and postinteraction condition groups within PD subjects (*p*>0.1 for all analyses).

**Table 5:** Significance Test for Coefficient of Variation of Stride Interval for PD and Healthy Subjects.

	PD Subjects						Healthy Subjects
	Preinteraction		Interaction		Postinteraction		Silent
PD Subjects							
Preinteraction	–		–		–		–
Interaction	*p* = 0.2		–		–		–
Postinteraction	*p* = 0.4		*p*>0.9		–		–
Healthy Subjects							
Silent	*p*<0.001	***	*p*<0.01	**	*p*<0.01	**	–

***p<0.001

**p<0.01.

Moreover, the relationship between the index values of disease severity and the rate of stride interval change (*β*) was investigated by focusing on the experimental results of the 26 subjects who contributed data for both the preinteraction and interaction conditions. The disease severity indexes were HY, UPDRS-2, and UPDRS-3. In each index, the 26 subjects with PD were divided into two subgroups whose index values of disease severity were higher and lower than the median index value.

The median HY index value was 3. There were nine subjects in the low HY subgroup and 17 in the high HY subgroup. Fig 3C and Tables 6 and 7 summarize the statistical comparison of the rate of change of stride interval (*β*) between these groups. There were interaction effects of the experimental conditions and the degree of HY on rate *β* (*F*(1,24) = 10.1, *p* = 0.05). In the preinteraction condition, rate *β* in the high HY subgroup (*M* ± *SD* = –2.62 × 10^−3^ ± 1.14 × 10^−3^) was not significantly different from that in the low HY subgroup (*M* ± *SD* = –2.48 × 10^−3^ ± 1.37 × 10^−3^) (*p*>0.9).

**Table 6:** Omnibus Analysis, Interaction Effects, and Post Hoc Comparisons for Change Ratio of Stride Interval for Mild and Severe PD Subjects.

Evaluation Index	Rating Scale	Severity (S)	Experimental Condition (C)		Analysis of Variance		
			PD subjects		Component	*F* value, *p* value	
			Preinteraction	Interaction			
Change Ratio of Stride Interval[×10^−3^]	Hoehn and Yahr Stage	Low	–2.40 ± 1.30	–0.384 ± 1.19	S:	*F*(1, 24) = 3.29, *p* = 0.08	
		High	–2.74 ± 1.11	–1.26 ± 1.83	C:	*F*(1, 24) = 32.4, *p*<0.001	***
					S*C:	*F*(1, 24) = 4.18, *p* = 0.05	*
	UPDRS Part 2	Low	–2.54 ± 1.17	0.118 ± 0.625	S:	*F*(1, 24) = 4.84, *p* = 0.05	*
		High	–2.71 ± 1.25	–1.59 ± 1.78	C:	*F*(1, 24) = 41.7, *p*<0.001	***
					S*C:	*F*(1, 24) = 7.16, *p* = 0.01	**
	UPDRS Part 3	Low	–2.56 ± 1.33	0.00318 ± 0.579	S:	*F*(1, 24) = 2.81, *p* = 0.1	
		High	–2.68 ± 1.12	–1.38 ±1.87	C:	*F*(1, 24) = 37.9, *p*<0.001	***
					S*C:	*F*(1, 24) = 4.36, *p* = 0.05	*

***p<0.001

**p<0.01

*p<0.05

**Table 7:** Significance Test for Change Ratio of Stride Interval for Mild and Severe PD Subjects in Hoehn and Yahr Stage.

PD Subjects	Low				High		
	Preinteraction		Interaction		Preinteraction		Interaction
Low							
Preinteraction	–		–		–		–
Interaction	*p*<0.001	***	–		–		–
High							
Preinteraction	*p*>0.9		*p*<0.001	***	–		–
Interaction	*p* = 0.2		*p* = 0.05	*	*p* = 0.03	*	–

***p<0.001

*p<0.05

However, in the interaction condition, rate *β* in the high HY subgroup (*M* ± *SD* = –1.33 × 10^−3^ ± 1.68 × 10^−3^) was significantly smaller compared with the low HY subgroup (*M* ± *SD* = 0.137 × 10^−3^ ± 0.751 × 10^−3^) (*p* = 0.05). Moreover, rate *β* in the preinteraction condition was significantly smaller than in the interaction condition in the low HY subgroup (*p*<0.001) and the high HY subgroup (*p* = 0.03).

The median UPDRS-2 index value was 10. One of the 26 subjects with PD did not have a UPDRS-2 diagnosis for clinical reasons. The remaining 25 were divided into subgroups with 12 in the low UPDRS-2 subgroup and 13 in the high UPDRS-2 subgroup. Fig 3D and Tables 6 and 8 summarize the statistical comparison in the rate of change of stride interval (*β*) between these groups. There were interaction effects of the experimental conditions and the degree of HY on rate *β* (*F*(1,23) = 7.16, *p* = 0.01). In the preinteraction condition, rate *β* in the high UPDRS-2 subgroup (*M* ± *SD* = –2.71 × 10^−3^ ± 1.25 × 10^−3^) was not significantly different from that in the low UPDRS-2 subgroup (*M* ± *SD* = –2.54 × 10^−3^ ± 1.17 × 10^−3^) (*p*>0.9). However, in the interaction condition, rate *β* in the high UPDRS-2 subgroup (*M* ± *SD* = –1.59 × 10^−3^ ± 1.78 × 10^−3^) was significantly smaller than in the low UPDRS-2 subgroup (*M* ± *SD* = –0.118 × 10^−3^ ± 0.625 × 10^−3^) (*p*<0.01). Moreover, in the low UPDRS-2 subgroup, rate *β* in the preinteraction condition was significantly smaller than in the interaction condition (*p*<0.001); in the high UPDRS-2 subgroup, rate *β* in the preinteraction condition was not significantly different from the interaction condition (*p* = 0.1).

**Table 8:** Significance Test for Change Ratio of Stride Interval for Mild and Severe PD Subjects in UPDRS Part 2.

PD Subjects	Low				High	
	Preinteraction		Interaction		Preinteraction	Interaction
Low						
Preinteraction	–		–		–	–
Interaction	*p*<0.001	***	–		–	–
High						
Preinteraction	*p*>0.9		*p*<0.001	***	–	–
Interaction	*p* = 0.3		*p*<0.01	**	*p* = 0.1	–

***p<0.001

**p<0.01

The median UPDRS-3 index value was 22. Again, the same subject with PD previously referred to did not have a UPDRS-3 diagnosis for clinical reasons. The remaining 25 subjects with PD were divided into UPDRS-3 subgroups with 11 in the low UPDRS-3 subgroup and 14 in the high UPDRS-3 subgroup. Fig 3E and Tables 6 and 9 summarize the statistical comparison in the rate of change of stride interval (*β*). There were interaction effects of the experimental conditions and the degree of UPDRS-3 on rate *β* (*F*(1,23) = 4.36, *p* = 0.05). In the preinteraction condition, rate *β* in the high UPDRS-3 subgroup (*M* ± *SD* = –2.68 × 10^−3^ ± 1.12 × 10^−3^) was not significantly different from that in the low UPDRS-3 subgroup (*M* ± *SD* = –2.56 × 10^−3^ ± 1.33 × 10^−3^) (*p*>0.9). Moreover, in the interaction condition, rate *β* in the high UPDRS-3 subgroup (*M* ± *SD* = –1.38 × 10^−3^ ± 1.87 × 10^−3^) was not significantly different from that in the low UPDRS-3 subgroup (*M* ± *SD* = –0.00318 × 10^−3^ ± 0.579 × 10^−3^) (*p* = 0.06). However, in the low UPDRS-3 subgroup, rate *β* in the preinteraction condition was significantly smaller than in the interaction condition (*p*<0.001). In contrast, in the high UPDRS-3 subgroup, rate *β* in the preinteraction condition was not significantly different from that in the interaction condition (*p* = 0.06).

**Table 9:** Significance Test for Change Ratio of Stride Interval for Mild and Severe PD Subjects in UPDRS Part 3.

PD Subjects	Low				High	
	Preinteraction		Interaction		Preinteraction	Interaction
Low						
Preinteraction	–		–		–	–
Interaction	*p*<0.001	***	–		–	–
High						
Preinteraction	*p*>0.9		*p*<0.001	***	–	–
Interaction	*p* = 0.1		*p* = 0.06		*p* = 0.06	–

***p<0.001

Next, to investigate the details of the relationship between the index values of disease severity and the rate of stride interval change (*β*), we also calculated the correlation coefficients for the 26 subjects who contributed data in both the preinteraction and interaction conditions. The disease severity indexes were HY, UPDRS-2, and UPDRS-3. Because one of the 26 subjects with PD did not have a UPDRS-2 diagnosis or a UPDRS-3 diagnosis for clinical reasons, the experimental results for the remaining 25 subjects with PD were used to calculate the correlations under UPDRS-2 and UPDRS-3.

Table 10 summarizes the correlation coefficients (*r*) between the index values of disease severity (HY, UPDRS-2, and UPDRS-3) and the rate of stride interval change (*β*) under the preinteraction and interaction conditions. In HY, the index showed a significant negative correlation with the rate *β* under the interaction condition (*r* = –0.465, *p* = 0.02) but not the rate *β* under the preinteraction condition (*r* = –0.169, *p* = 0.4). In UPDRS-2, the index showed a nonsignificant correlation with the rate *β* under both the preinteraction condition (*r* = –0.0104, *p*>0.9) and the interaction condition (*r* = –0.341, *p* = 0.09). In UPDRS-3, with the same profile of the index of HY, there was a significant negative correlation with the rate *β* under the interaction condition (*r* = –0.526, *p*<0.01) but not the rate *β* under the preinteraction condition (*r* = –0.151, *p* = 0.5).

**Table 10:** Correlation Coefficients (*r*) between the Index Values of Disease Severity (HY, UPDRS-2, and UPDRS-3) and the Rate of Stride Interval Change (*β*) under the Preinteraction Condition and the Interaction Condition.

PD Severity	Condition	Correlation Coefficient (*r*)	*p* value	
Hoehn and Yahr Stage	Preinteraction	–0.169	*p* = 0.4	
	Interaction	–0.465	*p* = 0.02	*
UPDRS Part 2	Preinteraction	–0.0104	*p*>0.9	
	Interaction	–0.341	*p* = 0.09	
UPDRS Part 3	Preinteraction	–0.151	*p* = 0.5	
	Interaction	–0.526	p<0.01	**

**p<0.01

*p<0.05

## Discussion

This study investigated the influence of interpersonal interaction using the interactive rhythmic cues generated by the Walk-Mate system on the festinating gait of subjects with PD, a disorder of gait rhythm generation. The clinical effect of interactive rhythmic cues was specifically addressed based on the experimental results.

The interactive rhythmic cues significantly affected the rate of change of stride interval (*β*) in the interaction condition group. Rate *β* of the interaction condition group was significantly larger and nearer zero than that of the preinteraction condition group. These results show that the involuntary acceleration of gait speed was smaller while walking with the interactive rhythmic cues. This indicates that the interactive rhythmic cues generated by the Walk-Mate system improved the festinating gait condition in subjects with PD. Thus, the interpersonal interaction between the PD subjects and the Walk-Mate system was effective for the recovery of, or compensation for, clinically disordered gait rhythm generation.

A previous study reported that an interpersonal interaction affected the fluctuation of stride interval as a typical indicator of intrapersonal dynamics in gait [9]. In that experiment, subjects with PD walked while listening to an interactive rhythmic cue, fixed-tempo rhythmic cue, and silence without any cues. Only the stride interval of the subjects walking with the interactive rhythmic cues showed 1/*f* fluctuation and the fluctuation characteristic was at the same level as the control subjects, although the stride interval of the subjects with PD did not show 1/*f* fluctuation before walking with the interactive rhythmic cues [4]. In contrast, the current study indicated that intrapersonal dynamics changing through interpersonal interaction was not limited to the stride interval fluctuation. Moreover, the current study showed that interpersonal interaction clinically improved festinating gait among subjects with PD. Therefore, interpersonal interaction may be effective for a variety of gait disorders associated with intrapersonal dynamics.

As shown in our experimental result (Fig 2), the interactive rhythmic cues significantly affected the rate of change of stride interval (*β*) in the postinteraction condition group. Rate *β* of the postinteraction condition group was significantly larger and nearer zero than that of the preinteraction condition group. This meant that the involuntary acceleration of gait speed was smaller when walking after receiving the interactive rhythmic cues. This suggests that the gait improvement effect for festinating gait of subjects with PD carried over after the interactive rhythmic cues generated by the Walk-Mate system had stopped. It was clarified that intrapersonal dynamics were changed by the interpersonal interaction between the subject and the Walk-Mate system, and these changes persisted after subjects became independent of the interpersonal interaction. This showed that the interpersonal interaction significantly affected relearning of the intrapersonal dynamics associated with clinically normal gait rhythm generation.

Another study reported that interpersonal interaction contributed to the relearning of intrapersonal gait dynamics [20]. In that experiment, subjects with PD received longitudinal gait training with interactive rhythmic cues, fixed-tempo rhythmic cues, 1/*f* fluctuating tempo rhythmic cues, and silence without any cues. Only the longitudinal gait training using interactive rhythmic cues gradually restored the stride interval fluctuation of the subjects to healthy 1/*f* levels. In contrast, training using the other conditions did not show a significant effect on the stride interval fluctuation of these subjects. The current study focused on festinating gait and clarified the carry-over effect of the interactive rhythmic cues based on interpersonal interaction. Thus, intrapersonal dynamics may promote the relearning associated with clinically normal gait rhythm generation that changed during interpersonal interaction in gait rehabilitation.

In general, a subject with PD shows varied disease severity depending on the progress of their symptoms. Did the difference in PD disease severity affect the influence of the interactive rhythmic cues on festinating gait? To address this question, the current study used disease severity indexes from common clinical diagnosis methods, namely HY, UPDRS-2, and UPDRS-3. The disease severity in HY is based on the assumption that the disease severity of PD patients is mostly significantly related to the severity of motor symptoms and loss of balance [27,40]. The disease severity in UPDRS-2 is based on activities of daily living. The disease severity in UPDRS-3 is based on motor examination. In other words, HY and UPDRS-3 directly reflect the patient’s motor function ability level. On the other hand, UPDRS-2 reflects relatively complex behaviors in a variety of daily situations. The relevance between the disease severity indices and the rate of change of stride interval (*β*) was then evaluated.

First, the subjects with PD belonging to the interaction condition group, which was the same as the preinteraction condition group, were divided into high and low disease severity groups based on HY, UPDRS-2, and UPDRS-3 and their rate *β* was compared. As shown in Fig 3C–3E, the interactive rhythmic cues were significantly effective for improving the festinating gait regardless of disease severity in each disease severity index; the improvement effects within the low disease severity group showed a tendency to be larger than within the high severity group, on each of the three disease severity indexes. Second, to explain further these relationships, the correlations between the rate *β* and the disease severities were also investigated. In Table 10, the disease severities of HY and UPDRS-3 showed significant negative correlations with the rate *β* under the interaction condition in PD subjects; however, there was not a significant correlation under the preinteraction condition. Nor was a significant correlation shown between the disease severity of UPDRS-2 and the rate *β*, regardless of experimental condition. This indicates that the improvement effect of the interactive rhythmic cues was larger at low disease severity of HY and UPDRS-3 and that PD subjects’ remaining motor ability showed a larger effect of gait improvement with interactivity.

These results support the proposition that interpersonal interaction with the PD subject using interactive rhythmic cues, as well as the gait rhythm of a therapist, is effective for improving generation of repetitive motor function such as human gait. According to the experimental results, the disease severity index of HY was negatively correlated with the degree of improvement. The HY score reflects motor ability, which is evaluated based on the body movement state, tremor, postural reflex, etc. The UPDRS-3 score also reflects aspects of motor ability and is relatively similar to HY; this evaluation is also based on the degree of paralytic symptoms of the lower and/or upper extremities, tremor, postural reflex, etc. A previous study indicated that high PD severity of movement ability is associated with weaker interaction in gait between rhythm generation in the nervous system and physical activity in the musculoskeletal system, based on gait environment [41]. According to this evidence, the subject with high PD severity has a lesser ability to use interpersonal interaction and the subject with low PD severity has a greater ability to use interpersonal interaction.

Therefore, integrating these experimental results with previous evidence, we suggest that PD subjects with a greater ability to use interpersonal interaction, which is associated with low disease severity, will show larger modification of festinating gait with interactive rhythmic cues than will PD subjects with high disease severity. This means that interpersonal interaction is related to the modification of festinating gait in subjects and is not in conflict with previous suggestions that interpersonal interaction establishes interactive rhythmic cues and changes gait dynamical characteristics or facilitates motor learning of the changed characteristics [9,20]. Thus, the interactivity between interpersonal rhythms in motor action is one of the essential factors in restoring motor action.

In rehabilitation, human interpersonal interaction is used to support daily activity and motor training. Such rehabilitation produces a cooperation effect between a therapist and a patient. This rehabilitation method draws on the patient’s inner abilities. Therefore, it is important to treat intrapersonal dynamics through interpersonal interaction.

What neural mechanism is affected in interpersonal interaction based on interactive rhythmic cues? Festinating gait was affected by interpersonal interaction in our current experiment and stride interval fluctuation was affected by interpersonal interaction in our previous experiment. The change in gait was improved from a disordered condition to a healthy level. This suggests that interpersonal interaction had an important role in assisting the subject to achieve sound gait. The Walk-Mate system uses the interpersonal synchronization of rhythms between the gait of subjects with PD and interactive rhythmic cues [9,15,42]; that is, a cross-feedback loop may be established through a temporal pattern [9,43,44]. Thus, the gait rhythm of the subject received input through cross-feedback from the external Walk-Mate system, and this cross-feedback loop contributed to the improvement in intrapersonal gait dynamics.

The rhythm modulation of human movement relates to the substantia nigra of the basal ganglia [24]. Patients with PD have a movement rhythm generation disorder resulting from dysfunction of this brain region [21–23]. Because the generation and modulation of movement rhythms were normalized by interpersonal interaction, it can be considered that the cross-feedback loop through rhythm between the subject and the system generating the interpersonal rhythmic cue activated the function of the basal ganglia or compensated for the disordered functionality of the basal ganglia.

In previous studies, human movement rhythm was stabilized by generating cooperative movement with other humans [7,45]. The synchronization process of rhythms between two individuals is deeply associated with the mechanism of stable rhythm generation. Although a subject with PD shows an unstable festinating gait when walking alone, they can execute a stabilized gait when interpersonal interaction with the interactive rhythmic cue of the Walk-Mate system is employed. Therefore, we suggest that the interpersonal interaction of rhythms between humans and the Walk-Mate system, and also between two humans, contribute not only to interpersonal stability, but also to rehabilitated gait rhythm generation through the cross-feedback of rhythm, using a temporal pattern. In the future, greater attention should be paid to interpersonal interaction and whether it is significantly effective for medical rehabilitation.

Finally, some limitations of the current study should be noted. Although the study clarified the clinical effect of interpersonal interaction, it was difficult to analyze scientifically longitudinal gait data among PD subjects with festinating gait due to the clinical features of festinating gait, and the physical strength and stamina of subjects. In some cases, the festinating gait itself, based on the clinical judgment of doctors and requests from the subjects, led to discontinuation of the task.

The spatial distance of trials varied slightly and was not recorded. Hence, we could not calculate speed and analyze the spatiotemporal gait features. However, this problem may be solved by expanding the development of multifaceted clinical application of interpersonal interaction for other gait disorders.

According to our experimental result shown in Fig 3 and Table 10, we suggest that the interpersonal interaction between rhythms in motor action contributes to and facilitates the restoration of motor action in disordered gait. However, the degree of improvement with the interpersonal interaction was significantly associated with activities of daily living, which represent more complex movement behaviors. For practical application, investigation with methods that link the effect of restoring the motor action and improving activities of daily living and quality of life is needed. Further social implementation of this technology in the future has the potential to expand the range of applications of interpersonal interaction to the rehabilitation and clinical domains.

## Supporting Information

S1 File Trial Protocol in Japanese Original Version. This file shows the original trial protocol of the experiment in this current article, written in Japanese.
(PDF)

S2 File Trial Protocol in English Translation Version. This file shows the English translation of the original trial protocol.
(PDF)

S3 File TREND Checklist. This file is the filled out TREND Checklist regarding this current article.
(PDF)
