# Supplementary material for: Effect of Interpersonal Interaction on Festinating Gait Rehabilitation in Patients with Parkinson’s Disease
Source: PLoS One. 2016 Jun 2;11(6):e0155540. doi: 10.1371/journal.pone.0155540 (PMC4890746; doi:10.1371/journal.pone.0155540)
Supplement: S1 File — This file shows the original trial protocol of the experiment in this current article, written in Japanese. (PDF) [file pone.0155540.s001.pdf]

研　究　計　画　書

1　研究課題

運動リズムの相互同調を活用するパーキンソン病患者の歩行支援手法

2　研究の概要

（1）　目　的

パーキンソン病における歩行の異常としては，歩幅の減少による小刻み歩行や，徐々に速足となる加速歩行，足が前に出なくなるすくみ足などがよく知られている．そして，このような歩行障害が生じるのは，中脳黒質からのドーパミン分泌が減少し，大脳基底核の機能不全を招き内的リズム生成の障害を生じることに原因があると考えられている．このような症状は投薬治療である程度は改善するものの，完全には消失せず，病気の進行により悪化することが知られている．そこで，これを補完する手法として，外的なリズム音刺激を患者に与えることによって内的リズム障害を代償し，安定したリズム形成を支援することで運動制御を改善することが期待される．このような背景から，本研究では，患者の歩行リズムと同調しやすいリズム音を提示し，歩行機能の改善を実現できる新しい運動支援技術の開発をめざす．

（2）　方　法

並んで歩いていると自然と歩調が揃うことは身近な経験であるが，共同研究者の三宅（東京工業大学）は，そのような人間同士の歩行リズムの同調現象（相互引き込み現象）に着目し，それを人間と歩行ロボットのあいだでの協調歩行として再現するシステム（Walk-Mate）の開発を進めてきた．具体的には，PC上に構成した仮想ロボットと人間の歩行リズムが，足接地タイミングに対応するリズム音を交換することで相互に歩調を同調させるシステムとして構成される．これまでに片麻痺や股関節疾患による歩行障害のリハビリテーションに活用され，運動の非対称性の緩和や歩行の安定性の向上などに有効であることが実証されてきた．本研究では，このような支援システムをパーキンソン病患者の歩行支援にはじめて活用するものである．具体的には，パーキンソン病の特徴的な症状のひとつである加速歩行に着目する．患者の単独歩行とWalk-Mate 歩行を時間的側面から比較し，Walk-Mate による加速歩行の制御性とそれによる加速抑制への効果を検証する．

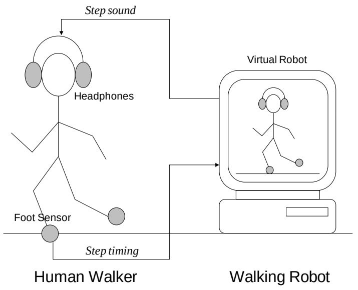

図 1　Walk-Mate システム

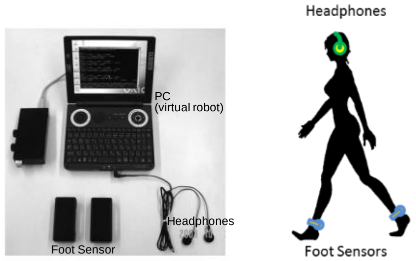

図 2　Walk-Mate 装着の様子

（3）　対　象

パーキンソン病の歩行障害の中でも加速歩行は転倒につながる可能性が高く，転倒から骨折，寝たきりに至る結果を招くことも多いとされる．このようなパーキンソン病での異常歩行を改善できればその恩恵は多大である．そこで，本研究では加速歩行の症状を示すパーキンソン病患者を対象とする．特に，歩行支援への有効性を評価する必要性から，姿勢反射障害があるが，主として単独歩行が可能なレベルの患者に協力を依頼する．

（4）　被験者の実体験（具体的に箇条書）

・ヘッドフォンから提示されるリズム音に合わせて自発的に歩行する
・靴に装着した歩行センサーによって歩行リズムのデータを記録する
・患者は上記の歩行支援システムを装着して廊下を数分間連続歩行する
・物理的な力を発生するような装置はいっさい患者の身体に装着しない
