# Supplementary material for: Effect of Interpersonal Interaction on Festinating Gait Rehabilitation in Patients with Parkinson’s Disease
Source: PLoS One. 2016 Jun 2;11(6):e0155540. doi: 10.1371/journal.pone.0155540 (PMC4890746; doi:10.1371/journal.pone.0155540)
Supplement: S2 File — This file shows the English translation of the original trial protocol. (PDF) [file pone.0155540.s002.pdf]

Study Plan

1 Study Theme

Gait Assist Method for Parkinson's disease patient based on interpersonal synchrony of movement rhythm

2 Summary of Study

(1) Purpose

In gait disorder of Parkinson's disease patient, the short-stepped gait which shows decreasing step length, the festinating gait which shows gradually decreasing stride interval, and the freezing of gait which shows hesitating foots' movement are well known. It is considered that these gait disorders are caused by decreasing dopamine secretion from a midbrain substantia nigra, the malfunction of basal ganglia is exhibited, and then an obstacle of the internal rhythm generation is developed. Although such a symptom is improved by medication treatment to some extent, the symptom is not completely restored and is worsened by progress of illness.

Therefore, the method for assisting the above medication treatment is expected that compensates the disordered internal rhythm generation with presentation of external rhythmic cue and improves motor control in gait with facilitating stable rhythm generation. Against the background, the purpose of this study was to develop a new motor assist technology for improving gait function by presentation of interactive rhythmic cue that is easy to synchronize the gait rhythm of the patient.

(2) Method

It is a familiar experience to synchronize gait steps between persons when walking together with someone. Co-researcher Dr. Miyake (Tokyo Institute of Technology) has been studying interpersonal synchronization (mutual entrainment) of gait rhythm between persons. He is developing the gait support system, named Walk-Mate, that emulates cooperative gait between a person and a virtual robot. In concreate, the virtual robot in Walk-Mate system, which is implemented in a computer, detects the person's step timing, synchronizes the robot's step timing to the detected person's step timing, and provides interactive rhythmic cue to the person.

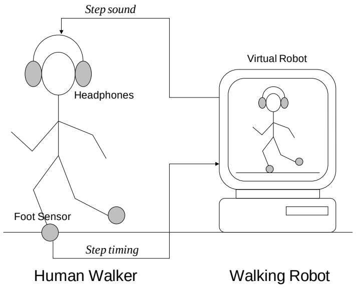

Fig 1. Walk-Mate system

Walk-Mate system is utilized in the rehabilitation of the gait disorder due to the hemiplegia and the hip osteoarthritis so far. An effect of Walk-mate system on improving the asymmetric property or gait stability of gait was clarified in previous studies.

This study utilizes such an assist system to facilitate the gait of Parkinson's disease patient for the first time. In concreate, the study focuses on festinating gait which is one of the pathognomonic symptoms. In the study, the independent gait of Parkinson's disease patient and the cooperative gait between the patient and Walk-Mate system are compared.

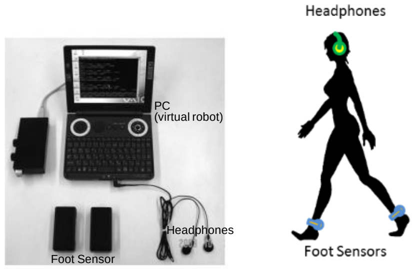

Fig 2. Experimental setup

(3) Subjects

The festinating gait is more likely to lead to a fall in a gait disorder of Parkinson's disease patients. Then, the festinating gait causes to break a bone from the fall, thereby leading to make the patients be bedridden. It is greatly beneficial for Parkinson's disease patients to develop the improvement method of gait disorder.

Therefore, the target subject is the Parkinson's disease patients with festinating gait in the study. Especially, in order to evaluate the effectiveness for gait assist, the study basically recruits the patient having the ability of walking alone.

(4) Clinical Study Protocol in Experiment

- Each subject walks with hearing rhythmic cue which is provided from headphones.
- The stride interval of the subject was detected by foot sensors attached under the shoes of the subject.
- The subject walks with wearing Walk-Mate system for a few minutes.
- The subject does NOT wear any other experimental systems which output physical forces generated by actuator.
